# Comparative Outcomes of Letrozole *Versus* Clomiphene Citrate for Ovulation Induction in Patients With PCOS: Systematic Review and Meta-Analysis

**DOI:** 10.5935/1518-0557.20250174

**Published:** 2026

**Authors:** Katherine Ann Reimão Miller, Gabriel Monteiro Pinheiro

**Affiliations:** 1 Medical Student, University of Santo Amaro, São Paulo, Brazil; 2 MD, Department of Obstetrics and Gynecology, University of Santo Amaro, São Paulo, Brazil

**Keywords:** polycystic ovary syndrome, PCOS, ovulation induction, letrozole, clomiphene citrate, infertility

## Abstract

**Objective:**

This study aimed to synthesize available high-quality randomized controlled trials (RCTs) comparing reproductive outcomes between letrozole (LE) and clomiphene citrate (CC) for ovulation induction in women with PCOS.

**Methods:**

Following PRISMA guidelines, this review was registered in PROSPERO (CRD420251013416). A comprehensive search was conducted in PubMed, Cochrane Library, Elsevier, the National Library of Medicine, and Google Scholar up to March 2025. A total of 32 RCTs were included. Statistical analysis used a random-effects model to calculate risk ratios (RR) with 95% confidence intervals (CI), as determined using Review Manager. Heterogeneity was assessed with the I^2^ statistic. The risk of bias was evaluated using the ROB 2 tool. The primary outcome was ovulation rate; secondary outcomes included pregnancy, live birth, miscarriage, and multiple pregnancy rates.

**Results:**

LE showed higher ovulation (RR: 1.18; 95% CI: 1.11-1.25; I^2^: 57%) and pregnancy rates (RR: 1.57; 95% CI: 1.39-1.76; I^2^: 21%) compared to CC. LE also had a higher live birth rate (RR: 1.54; 95% CI: 1.24-1.91; I^2^: 35%). Miscarriage rates were similar between groups (RR: 0.97; 95% CI: 0.73-1.28; I^2^: 0%). The multiple pregnancy rate was significantly lower in the LE group (RR: 0.42; 95% CI: 0.18-0.98; I^2^: 0%).

**Conclusions:**

Letrozole shows greater effectiveness than clomiphene citrate for ovulation induction in women with PCOS, with a lower risk of multiple pregnancy and similar miscarriage risk. These findings support its use as a first-line treatment in clinical practice.

## INTRODUCTION

Polycystic ovary syndrome (PCOS) is a chronic and heterogeneous endocrine disorder that affects between 8-20% of women of reproductive age each year. It is also known as hyperandrogenic chronic anovulation or Stein-Leventhal syndrome (Singh *et al*., 2023; El Hayek *et al*., 2016; Witchel *et al*., 2019). PCOS can occur at any age but most commonly begins between 18 and 30 years old (Bremer, 2010). Clinically, PCOS presents with menstrual irregularities, anovulatory infertility, hirsutism, acne, obesity, alopecia, and metabolic problems (Legro *et al*., 1999).

For many years, PCOS was diagnosed using the Rotterdam criteria, established in 2004, which required at least two of the following three features: I. Oligoor anovulation; II. Clinical and/or biochemical signs of hyperandrogenism; and III. Polycystic ovarian morphology on ultrasound, defined as ≥12 follicles measuring 2-9 mm in diameter and/or ovarian volume >10 mL in at least one ovary (Rotterdam, 2004). However, in 2023, the International Evidence-Based Guideline for Assessment and Management of PCOS introduced important updates to these criteria. While the overall structure-requiring two of three features-was maintained, key changes were made: I. The follicle count threshold was increased from ≥12 to ≥20 follicles per ovary. II. Either serum anti-Müllerian hormone (AMH) or ultrasound may be used to define PCOS. III. If irregular menstrual cycles and hyperandrogenism are present, diagnosis is simplified, and ultrasound or AMH are not required for diagnosis (Teede *et al*., 2023).

The pathophysiology of PCOS is driven by interconnected endocrine and metabolic disturbances. Excess androgen production from the ovaries and adrenal glands contributes to hyperandrogenism, which disrupts hypothalamic GnRH pulse frequency and promotes the growth of primordial and antral follicles (Ibáñez *et al*., 2017). The dysregulation of GnRH frequency increases LH relative to FSH. The elevated LH:FSH ratio promotes theca cell hyperplasia and follicular fluid accumulation, forming cystic structures along the ovarian periphery. Many follicles, mostly pre-antral and antral, become arrested, leading to excessive androgen production and perpetuating the hormonal imbalance (Walters *et al*., 2018; Bulsara *et al*., 2021; Ashraf *et al*., 2019).

Due to the complex etiology of PCOS, its treatment often combines multiple approaches, especially to address the most troublesome symptoms such as irregular periods, hirsutism, and anovulatory infertility. Anovulation in PCOS is linked to low FSH levels and the halt of antral follicle growth during its final maturation stages (Singh *et al*., 2023). Ovulation induction is an effective treatment for women with PCOS who want to conceive. For more than 40 years, the first-line drug for ovulation induction has been clomiphene citrate (CC), a selective estrogen receptor modulator. Clomiphene citrate (CC) mimics estrogen and binds to estrogen receptors (ER) in the reproductive system, but unlike estrogen, it binds to nuclear ERs for an extended period, reducing ER levels by blocking their regeneration. Its primary role in inducing ovulation is through its action at the hypothalamus. CC lowers hypothalamic ERs, making circulating estrogen seem low, which prompts the body to compensate by changing gonadotropin-releasing hormone (GnRH) secretion. This results in increased release of pituitary gonadotropins, which stimulate ovarian follicle growth.

However, in 2023, new recommendations for PCOS treatment were included in the international evidence-based guideline for assessing and managing PCOS, published in the Fertility and Sterility Journal (Teede *et al*., 2023). These guidelines show that letrozole (LE), an aromatase inhibitor, has been accepted as the new first-line medication for ovulation induction in women with PCOS and anovulatory infertility. Letrozole (LE) is a selective oral aromatase inhibitor that binds to the P450 aromatase enzyme, blocking the conversion of testosterone to estradiol and androstenedione to estrone. Its exact mechanism for inducing ovulation is not fully understood, but it is believed to act through both central and peripheral pathways. Centrally, letrozole lowers estrogen levels, preventing negative feedback on the hypothalamic-pituitary-gonadal (HPO) axis. Peripherally, it inhibits the conversion of androgens to estrogen, causing a temporary buildup of androgens in the ovaries. This buildup enhances follicular sensitivity by increasing FSH receptor expression and may also stimulate insulin-like growth factor 1 (IGF-1) and other factors that promote folliculogenesis (Yang *et al*., 2021).

Although recent clinical guidelines have increasingly favored LE as the first-line agent for ovulation induction in PCOS, a systematic review with meta-analysis is still needed to synthesize the growing body of evidence and provide clearer guidance for clinical practice. Individual randomized trials vary in sample size, patient populations, dosing protocols, and reported outcomes, which may limit the generalizability of their findings. By pooling data from randomized trials and analyzing multiple clinically relevant endpoints, this study aims to address these limitations and offer a comprehensive, statistically powered comparison of LE and CC. Such synthesis is crucial to identify the most effective and safe treatment options, reduce practice variability, and support evidence-based decision-making in reproductive medicine.

## MATERIAL AND METHODS

This systematic review and meta-analysis was conducted following the PRISMA guidelines (Moher *et al*., 2009). The protocol was registered with the International Prospective Register of Systematic Reviews (PROSPERO Registration Number: CRD420251013416).

The literature search strategy for the meta-analysis was conducted by both authors: one independent researcher (Miller, K.) collected 701 studies from multiple electronic databases (PubMed, Cochrane Library, National Library of Science, Elsevier) and other journals from different countries around the world. Later, another independent researcher (Pinheiro, G.) independently reviewed the studies. Predefined combinations of keywords were used: “PCOS,” “polycystic ovary syndrome,” “infertility,” “ovulation induction,” “aromatase inhibitors,” “letrozole,” “clomiphene citrate.” The search strategy was expanded by screening reference lists of eligible studies, using the ‘related articles’ feature in electronic databases, and manually consulting international medical journals. No restrictions on publication date or country were applied, but only studies in English were included.

This meta-analysis included only randomized controlled trials (RCTs) comparing LE versus CC alone, used for ovulation induction in women with PCOS, and reporting at least one of the outcomes included in this meta-analysis. Inclusion criteria were women aged 18 to 40 years old, diagnosed with PCOS according to the Rotterdam criteria (Rotterdam, 2004), presenting at least two of three variables: oligo-ovulation and/or anovulation; clinical or biochemical signs of hyperandrogenism; and polycystic ovaries confirmed by ultrasound, having more than 12 follicles smaller than 10 mm in diameter or increased ovarian volume greater than 10 cm^3^ in one or both ovaries, and at least one patent fallopian tube confirmed via HSG. Only studies investigating ovulation induction in natural menstrual cycles were included. Exclusion criteria were: (i) patients with uncontrolled endocrine disorders; (ii) BMI greater than 35 kg/m^2^; (iii) patients who have used other medications for ovulation induction besides LE or CC during the specified period.

The primary outcome of this study was ovulation rate per patient, determined by ultrasound or mid-luteal progesterone levels. The secondary outcomes included: (i) pregnancy rate per patient, defined as the visualization of one or more gestational sacs and a positive β-hCG test; (ii) live birth rate per patient; (iii) miscarriage rate per patient, defined as the loss of clinical pregnancy before 20 weeks of gestation; (iv) multiple pregnancy rate per patient.

**Figure 1:**
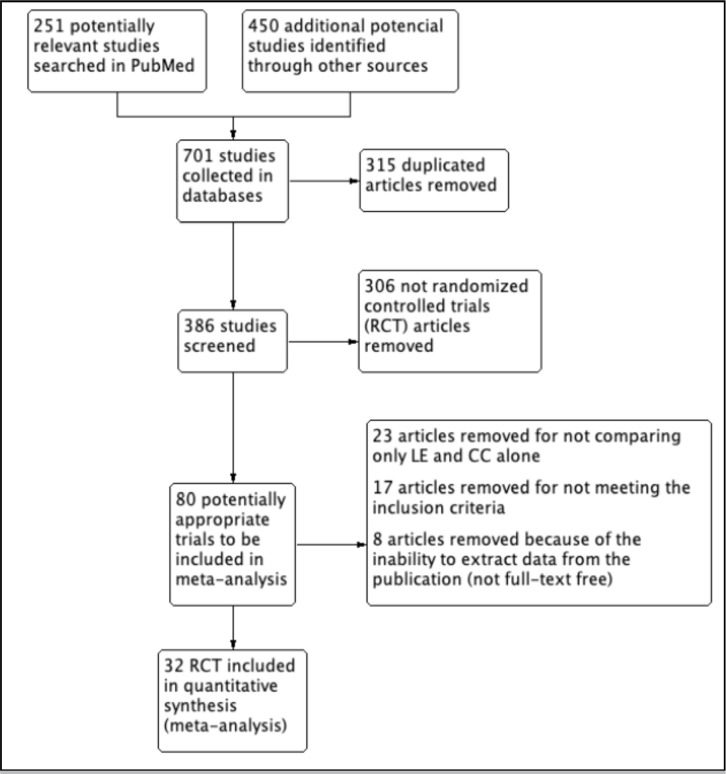
PRISMA Flow diagram of search strategy and study selection.

The study selection was done independently by one reviewer (Miller, K.), starting with database searches, specifically in PubMed, where 251 potentially relevant studies were identified. Subsequently, 450 additional studies were collected from other databases such as the National Library of Science, Cochrane Library, Elsevier, and Google Scholar. Both authors then performed data extraction and screening for the 701 studies collected, excluding 315 duplicates and 306 non-RCT articles. The remaining 80 potentially suitable trials were reanalyzed, resulting in the removal of 23 articles for not comparing only LE and CC, 17 for not meeting inclusion criteria, and 8 for the inability to extract data from the publication. Therefore, this study included 32 RCTs in the quantitative synthesis (meta-analysis). The flow diagram of the search strategy and study selection is shown in Figure 1.

**Figure 2:**
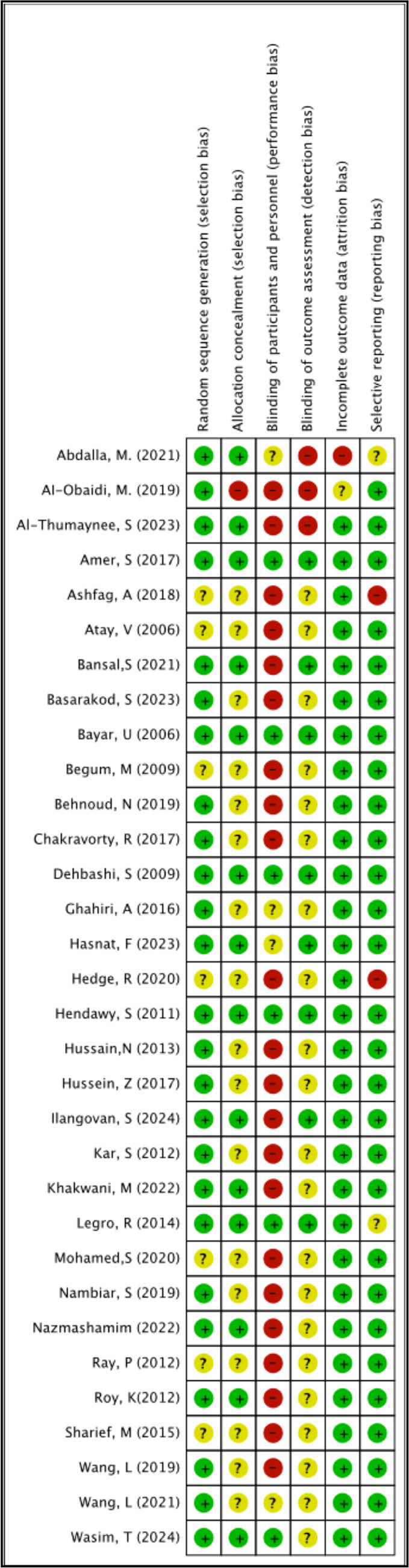
Risk of bias summary + (color green): low risk of bias? (color yellow): unclear risk of bias- (color red): high risk of bias.

Risk of bias in individual studies was assessed using the Risk of Bias table in Review Manager 5.4.1 from Cochrane. The biases were evaluated based on the five domains of the revised Cochrane Risk of Bias 2 tool (Sterne *et al*., 2019): random sequence generation, allocation concealment, blinding of participants and personnel, blinding of outcome assessment, incomplete outcome data, and selective reporting bias. Each domain was judged as either low, unclear, or high risk of bias. For studies considered high risk in one or more domains, the confidence in their results was substantially lowered. In this study’s perspective, the risk of bias summary is presented in Figure 2.

Statistical analysis and risk of bias summaries were conducted using Review Manager 5.4.1 software (RevMan, version 5.4; The Cochrane Collaboration, 2020). A random effects model was employed for the meta-analysis. For dichotomous data and primary outcomes, forest plots were generated using the Mantel-Haenszel method and risk ratios (RR) as effect measures. The analysis was performed with 95% confidence intervals (CI) and a total confidence interval set at 95%, on a 100.00 scale. Heterogeneity (*I*^2^) was included as a measure of consistency.

## RESULTS

We included 32 RCTs from 11 different countries: Iraq (4), Egypt (3), the United Kingdom (1), Pakistan (3), Turkey (2), India (10), Bangladesh (2), Iran (3), Malaysia (1), the United States (1), and China (2). A total of 4858 patients were enrolled, with 2,297 in the letrozole group (LE) and 2561 in the clomiphene citrate group (CC). The number of cycles performed until pregnancy and/or ovulation was reported in 22 of the 33 RCTs, totaling an average of 9802 cycles, with 4798 in the LE group and 5004 in the CC group. In 30 studies, the drugs were administered from day 3 to day 7 of the menstrual cycle. Two studies administered the drugs from day 2 to day 6, and one study administered the drugs for only four days, from day 5 to day 9 of the cycle. HCG was administered at doses of 5 to 10,000 IU to trigger ovulation when at least one follicle was ≥15-22mm. Ultrasound (USG-TV) was performed serially to assess follicular development, and pregnancy was confirmed via β-hCG test and ultrasound. The characteristics of the included studies are listed in Table 1.

**Table 1:** Characteristics of the included studies. LE=letrozole; CC=clomiphene citrate; n= number.

Author (year)	Country	Intervention	Method of Conception	Ovulation triggering and hCG use	Patients (n)	Cycles (n)	Outcomes included in the meta-analysis
**Ahmed Abdalla *et al*. (2021)**	Egypt	-LE: 5 mg/day for 5 days (3^rd^-7^th^ day of the cycle)-CC: 100mg/day for 5 days (3^rd^-7^th^ day of the cycle)	Timed sexual intercourse	LE: 35(95%) received hCG 10.000UI (IM) when at least one follicle >18mm CC: 24(80%) received hCG 10.000UI (IM) when at least one follicle >18mm ^*^Both patients’ groups were advised to have intercourse 24 to 36 hours after hCG injection	LE: 40 CC: 40	LE:120 CC:120	• Pregnancy rate • Ovulation rate
**Al-Obaidi *et al*. (2019)**	Iraq	-LE: 2.5mg 2x/day for 5 days (3^rd^-7^th^ day of the cycle) -CC: 50mg 2x/day for 5 days (3^rd^-7^th^ day of the cycle)	Timed sexual intercourse	Both groups received hCG subcutaneously, when at least one follicle >18mm, to trigger ovulation	LE: 40 CC: 40	N/R	• ovulation rate
**Al-Thuwaynee & Swadi (2023)**	Iraq	-LE: 2,5 - 7,5 mg (stair-step pattern) for 5 days (2^nd^-6^th^ day) -CC: 50 - 150 mg^*^(stair-step pattern) for 5 days (2^nd^-6^th^ day)	Timed sexual intercourse	Both groups received hCG, when at least one follicle >18mm, to trigger ovulation	LE: 50 CC: 50	LE: 150 CC: 150	• Pregnancy rate • Ovulation rate
**Amer *et al*.** **(2017)**	United Kingdom	-LE: 2,5 - 5 mg/day^*^ (increased in the next cycle in non-responders) starting on days 2-4 of the cycle -CC: 50-100 mg^*^	Timed sexual intercourse	Ovulation was diagnosed with a progesterone level of ≥25 nmol/l, a follicle diameter ≥17 mm, and/or occurrence of pregnancy. Cycles were initially monitored with ultrasound follicle tracking. The study does not explicitly state the administration of exogenous hCG as a trigger.	LE: 80 CC: 79	LE: 320 CC: 474	• Pregnancy rate • ovulation rate • live birth rate • miscarriage rate • multiple pregnancy rate
**Ashfaq *et al.*** **(2018)**	Pakistan	-LE: 5 mg/day for 5 days -CC: 100 mg/day for 5 days	did not mention timed intercourse or IUI	Induction of ovulation is assessed by TVS. If follicle of >2cm is found on 12 days TVS and smaller/collapsed on 16 days TVS, ovulation induction was labeled.	LE: 40 CC: 40	N/R	• ovulation rate
**Atay *et al.*** **(2006)**	Turkey	-LE: 2,5 mg for 5 days (3^rd^-7^th^ day) -CC: 100 mg/day for 5 days (3^rd^-7^th^ day	Timed sexual intercourse	Both groups received hCG 10.000UI subcutaneously (SC), when at least one follicle ≥ 18 mm, to trigger ovulation	LE: 51 CC: 55	LE: 51 CC: 55	• Pregnancy rate • ovulation rate • multiple pregnancy rate
**Banerjee Ray et al. (2012)**	India	-LE: 2,5 mg/day for 5 days (3rd-7th day of the cycle) -CC: 100 mg/day for 5 days (3rd-7th day of the cycle)	did not mention timed intercourse or IUI	A single injection of 10,000 IU hCG was given, if at least one follicle attained 18 mm.	LE: 69 CC:78	LE:132 CC:156	• Pregnancy rate • ovulation rate
**Bansal *et al*.** **(2021)**	India	-LE: 2,5 - 7,5 mg/day for 5 days (2^nd^-6^th^ day) the dose was increased by 2.5mg every cycle -CC: 50-150 mg/day for 5 days (2^nd^-6^th^ day) the dose was increased by 50 mg every cycle	Timed sexual intercourse	When the size of the dominant follicle reached >18 mm, human Chorionic Gonadotrophin (hCG) 5000 IU was given as a trigger intramuscularly for ovulation.	LE: 45 CC:45	LE: 135 CC:135	• Pregnancy rate • ovulation rate
**Basarakod** ***et al*. (2023)**	India	-LE: 2,5-7,5mg/day for 5 days (2^nd^-6^th^ day) (stepwise increase in dose up to 7.5mg in the next cycle in the absence of ovulation) -CC: 50 - 150 mg for 5 days (2^nd^-6^th^ day)	Timed sexual intercourse	When a matured follicle of size 18-22 mm was noted, ovulation trigger using HCG 10000IU injection IM was given	LE: 40 CC: 40	LE: 80 CC:104	• Pregnancy rate • ovulation rate
**Bayar et al.** **(2006)**	Turkey	-LE: 2,5 mg/day for 5 days (3rd-7^th^ day of the cycle) -CC: 100 mg/day for 5 days (3^rd^-7^th^ day of the cycle)	Timed sexual intercourse	Human chorionic gonadotropin (hCG) 10,000 IU, SC, was administered to trigger ovulation when at least one mature follicle (≥18 mm) developed.	LE: 38 CC: 36	LE: 99 CC: 95	• Pregnancy rate • ovulation rate • miscarriage rate
**Begum et al.** **(2009)**	Bangladesh	-LE: 7,5 mg/day for 5 days (3^rd^-7^th^ day of the cycle) -CC: 150 mg/day for 5 days (3^rd^-7^th^ day of the cycle)	Timed sexual intercourse	A single injection of 10,000 IU hCG was given if at least one follicle attained 18 mm. Ovulation was ascertained by observing rupture of the follicle by ultrasonogram (USG) and day 21 serum P level (≥10ng/mL was considered ovulatory).	LE: 32 CC: 32	LE: 64 CC: 64	• Pregnancy rate • ovulation rate • miscarriage rate • multiple pregnancy rate
**Behnoud** **et al.** **(2019)**	Iran	-LE: 5 mg/day for 5 days (3^rd^-7^th^ day of the cycle) -CC: 100mg/day for 5 days (3^rd^-7^t^h day of the cycle)	Timed sexual intercourse	hCG was prescribed for LH surge if there was 1 or more than one dominant follicle with triple line endometrial pattern.	LE: 40 CC: 40	N/R	• Pregnancy rate
**Chakravorty et al.** **(2017)**	India	-LE: 2,5 - 5 mg^*^ for 5 days (3^rd^-7^th^ day of the cycle) -CC: 50-150 mg^*^ for 5 days (3^rd^-7^th^ day of the cycle)	Timed sexual intercourse	When at least one mature follicle (mean diameter ≥18mm) was observed, 10,000IU of human chorionic gonadotropin (hCG) were given subcutaneously to trigger ovulation.	LE: 66 CC: 61	LE: 198 CC: 183	• Pregnancy rate • ovulation rate
**Dehbashi** **et al.** **(2009)**	Iran	-LE: 5 mg/day for 5 days (3^rd^-7^th^ day of the cycle) -CC: 100 mg/day for 5 days (3^rd^-7^th^ day of the cycle)	Timed sexual intercourse	Intramuscular hCG (10,000IU) was administered to trigger ovulation when at least one mature follicle (≥18mm) was developed.	LE: 50 CC: 50	N/R	• Pregnancy rate • ovulation rate • live birth rate • miscarriage rate • multiple pregnancy rate
**Ghahiri *et al*.** **(2016)**	Iran	-LE: 5 mg/day for 5 days (3^rd^-7^th^ day of the cycle) -CC: 100 mg/day for 5 days (3^rd^-7^th^ day of the cycle)	Timed sexual intercourse	Ovulation confirmation relied on calendar, history-taking, and pregnancy occurrence. In case of delayed menstruation in a patient who had ovulated, β-HCG was measured to confirm pregnancy.	LE: 50 CC: 51	N/R	• Pregnancy rate • ovulation rate • miscarriage rate • multiple pregnancy rate
**Hasnat et al.** **(2023)**	Bangladesh	-LE: 2,5 - 7,5 mg/day for 5 days (2nd-6th day) the dose was increased by 2.5mg every cycle -CC: 50-150 mg/day for 5 days (2nd-6th day) the dose was increased by 50 mg every cycle	Timed sexual intercourse	Once the dominant follicle surpassed 18mm in size, ovulation was induced by administering 5000IU of hCG intramuscularly.	LE: 51 CC: 51	LE: 102 CC: 102	• Pregnancy rate • ovulation rate
**Hegde &** **Maitra** **(2020)**	India	-LE: 2,5 mg/day for 5 days (3^rd^-7^th^ day of the cycle) -CC: 100 mg/day for 5 days (3^rd^-7^th^ day of the cycle)	Timed sexual intercourse	hCG was administered at a dose of 5000 IU when at least 1 mature follicle (18-22mm) was detected.	LE: 25 CC: 25	LE: 25 CC: 25	• Pregnancy rate • ovulation rate • multiple pregnancy rate
**Hendawy** **et al.** **(2011)**	Egypt	-LE: 2,5 mg/day for 5 days (3^rd^-7^th^ day of the cycle) -CC: 100 mg/day for 5 days (3^rd^-7^th^ day of the cycle)	Intrauterine insemination (IUI) was performed 32-36 hours after hCG. A single insemination was performed. Semen specimens were prepared using Ham’s F-10 medium with L-glutamine, washed, and kept at room temperature for 45 minutes until transfer into the uterine cavity using a Wallace intrauterine insemination catheter. Time interval between semen production and transfer did not exceed 2.5 hours.	HCG 10,000 IU was given intramuscularly to trigger ovulation when at least one mature follicle ≥18mm diameter was detected. Luteal Phase Support: Progesterone vaginal pessaries 400 mg once daily for 15 days.	LE: 30 CC: 30	N/R	• Pregnancy rate • multiple pregnancy rate
**Hussain et al.** **(2013)**	Malaysia	-LE: 5 mg/day for 5 days (5th-9th day of the cycle) -CC: 100 mg/day for 5 days (5^th^-9^th^ day of the cycle)	did not mention timed intercourse or IUI	Ovulation was diagnosed when the mature dominant follicle (DF) was approximately 18 to 22mm, followed by evidence of rupture approximately 3 to 4 days later, observed via serial transvaginal ultrasound (TVS). hCG use was not mentioned.	LE: 75 CC: 75	LE: 75 CC: 75	• Pregnancy rate • ovulation rate
**Hussein *et al*.** **(2017)**	Iraq	-LE: 5 mg/day for 5 days (5^th^-9^th^ day of the cycle) -CC: 100 mg/day for 5 days (5^th^-9^th^ day of the cycle)	Timed sexual intercourse	Recombinant hCG was administered subcutaneously to trigger ovulation when at least one mature follicle 18 mm diameter was detected.	LE: 40 CC: 40	LE: 40 CC: 40	• Pregnancy rate
**Ilangovan (2024)**	India	-LE: 2,5 mg/day for 5 days (3^rd^-7^th^ day of the cycle) the dose was increased in non-responders up to 7.5mg/day in the next cycle -CC: 50mg/day for 5 days (3^rd^-7^th^ day of the cycle) the dose was increased in non-responders up to 150 mg in the next cycle	Timed intercourse/intrauterine insemination (IUI) as appropriate. The article does not explicitly state the exact number of patients who underwent IUI neither the methods	An injection of 5,000 to 10,000 IU of human chorionic gonadotropin (hCG) was administere	LE: 192 CC: 192	LE: 192 CC: 192	• Pregnancy rate • ovulation rate • live birth rate • miscarriage rate
**Kar (2012)**	India	-LE: 5 mg/day for 5 days (2^nd^-6^th^ day) -CC: 100 mg/day for 5 days (2^nd^-6^th^ day)	Timed intercourse or Intrauterine Insemination (IUI) was advised. The choice between both was made “as per the patient requirement”	A single injection of 10,000 IU of hCG was administered, if at least one follicle attained 17-18 mm diameter during follicular monitoring. Luteal Support: Duphastone 10 mg daily was given to all patients.	LE: 52 CC: 51	LE: 52 CC: 51	• Pregnancy rate • ovulation rate • live birth rate • miscarriage rate • multiple pregnancy rate
**Khakwani** ***et al*.** **(2022)**	Pakistan	LE: 5 mg/day for 5 days (3^rd^-7^th^ day of the cycle) CC: 100 mg/day for 5 days (3^rd^-7^th^ day of the cycle)	did not mention timed intercourse or IUI	All women underwent transvaginal scan (TVS) for the evaluation of ovulation induction. If follicle of more than 2 cm was located on 12 day on TVS and/or small/collapsed on 16 day on TVS, ovulation induction was considered yes or otherwise.	LE: 36 CC: 34	N/R	• ovulation rate
**Legro *et al*.** **(2014)**	United States	After spontaneous menses or withdrawal bleeding induced by progestin administration progesterone acetate: -LE: 2,5 mg/day for 5 days (3^rd^-7^th^ day of the cycle) the dose was increased in non-responders up to 7.5mg/day in the next cycle (stepwise increase dose) -CC: 50 mg/day for 5 days (3^rd^-7^th^ day of the cycle) the dose was increased in non-responders up to 150 mg in the next cycle	Timed intercourse and Intrauterine Insemination (IUI)	Ovulation predictor kits were not used.	LE: 301 CC: 308	LE:1352 CC:1425	• Pregnancy rate • ovulation rate • live birth rate • miscarriage rate • multiple pregnancy rate
**Mohamed** ***et al*.** **(2020)**	Egypt	-LE: 5 mg/day for 5 days (3^rd^-7^th^ day of the cycle) -CC: 100 mg/day for 5 days (3^rd^-7^th^ day of the cycle)	did not mention timed intercourse or IUI	If a dominant follicle (DF) was present (DF > 12 mm), a repeat TVS was performed every 2 days. Ovulation was diagnosed when the mature DF was approximately 18 to 22 mm followed by evidence of rupture approximately 3 to 4 days later. If a dominant follicle (DF) was absent (DF < 12 mm), a repeat TVS was performed every 3 - 4 days later.	LE: 50 CC: 50	LE: 152 CC: 156	• Pregnancy rate • ovulation rate
**Nambiar** **(2019)**	India	Controlled ovarian hyperstimulation (COH): -LE: 5 mg/day for 5 days (3^rd^-7^th^ day of the cycle) -CC: 100 mg/day for 5 days (3^rd^-7^th^ day of the cycle)	Timed sexual intercourse	A single injection of hCG 10,000 IU IM was given if at least one follicle was >14 mm and the endometrial thickness at least more than 8 cm. A second TVS was done after 48 hours of hCG to observe the release of the egg.	LE: 104 CC: 96	N/R	• Pregnancy rate • ovulation rate • miscarriage rate
**Nazmashamim *et al*.** **(2022)**	India	LE: 5 mg/day for 5 days (2^nd^-6^th^ day) CC: 100 mg/day for 5 days (2^nd^-6^th^ day)	did not mention timed intercourse or IUI	hCG 5000 IU was administered within 24 hrs after the appearance of the dominant follicle	LE: 32 CC: 31	N/R	• Pregnancy rate • ovulation rate
**Roy *et al*. (2012)**	India	-LE: 2,5mg/day for 5 days (3^rd^-7^th^ of the cycle). The dose would be increased up to 5mg/day in the next cycle in non-responders -CC: 50mg /day for 5 days (3^rd^-7^th^ day of the cycle). The dose would be increased up to 100mg/day in non-responders	Timed sexual intercourse	Injection hCG 10,000 IU intramuscularly was given to the patients when a dominant follicle ≥18 mm and ET ≥6 mm was observed.	LE: 98 CC: 106	LE: 94 CC: 18	• Pregnancy rate • ovulation rate • live birth rate • miscarriage rate
**Sharief & Nafee** **(2015)**	Iraq	-LE: 2,5 - 5 mg^*^ for 5 days (3^rd^-7^th^ day of the cycle) -CC: 100 - 200 mg^*^ for 5 days (3^rd^-7^th^ day of the cycle)	Timed sexual intercourse	When at least one mature follicle (mean diameter >18 mm) was observed, 10.000IU of human chorionic gonadotropin (HCG) was given subcutaneously to trigger ovulation.	LE: 120 CC: 119	N/R	• Pregnancy rate • ovulation rate
**Wang *et al*.** **(2019)**	China	-LE: 2,5 mg/day for 5 days (3^rd^-7^th^ day of the cycle) -CC: 50 mg/day for 5 days (3^rd^-7^th^ day of the cycle)	Timed sexual intercourse	When at least one dominant follicle reached 18mm in diameter, human chorionic gonadotropin (hCG) at a dose of 8000 or 10,000IU was given intramuscular to trigger ovulation.	LE: 120 CC: 119	N/R	• Pregnancy rate • ovulation rate
**Wang *et al*.** **(2021)**	China	-LE: 2,5 mg/day for 5 days (5^th^-9^th^ day of the cycle) -CC: 50 mg/day for 5 days (5^th^-9^th^ day of the cycle)	did not mention timed intercourse or IUI	Human chorionic gonadotropin (hCG) at a dose of 5000-10000IU was used to trigger ovulation when a dominant follicle appeared (the average diameter ≥18 mm).	LE: 90 CC:90	LE: 90 CC: 90	• Pregnancy rate • ovulation rate • live birth rate • miscarriage rate • multiple pregnancy rate
**Wasim *et al*. (2024)**	Pakistan	-LE: 2,5mg/day for 5 days (2^nd^-6^th^ day of the cycle). The dose would be increased up to 7.55mg/day in the next cycle in non-responders -CC: 50mg/day for 5 days (2^nd^-6^th^ day of the cycle). The dose would be increased up to 150mg/day in the next cycle in non-responders	Timed sexual intercourse	When at least one ovarian follicle achieved a diameter of 18-25 mm, then injection HCG 10,000 IU IM was given.	LE: 110 CC:110	LE: 550 CC: 550	• Pregnancy rate • ovulation rate

The process of random sequence generation was satisfactory in twenty-six studies and unclear in seven studies. Allocation concealment was adequate in fourteen studies, inadequate in one study, and unclear in the remaining studies. The blinding of participants and personnel posed the highest risk of bias among the five domains, with twenty-three studies rated as inadequate, six as adequate, and four as unclear. The blinding of outcome assessment was the domain with the most unclear ratings, with twenty-two studies unclear, eight adequate, and only three rated as inadequate. Incomplete outcome data was generally adequate, with only one study rated as unclear, one as inadequate, and the remaining thirty-one as adequate. Overall, selective reporting was considered adequate, with twenty-nine studies rated as adequate, two as unclear, and two as inadequate. The risk of bias summary can be seen in Figure 2.

The ovulation rate per patient randomized was assessed as the primary outcome of this study and was reported in 28 RCTs, involving a total of 4198 women (LE group: 2095; CC group: 2103). There were 1,590 ovulation events in the LE group compared to 1,347 in the CC group. The pooled analysis showed a statistically significant benefit for letrozole over clomiphene citrate in inducing ovulation. The risk ratio (RR) was 1.1816 (95% CI: 1.1094 to 1.2586; *p*<0.00001), indicating that women treated with letrozole had an 18% greater chance of ovulating than those receiving clomiphene citrate.

**Figure 3:**
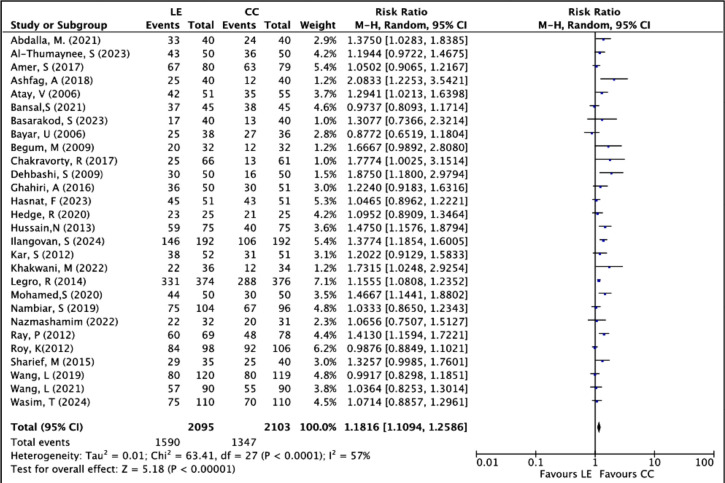
Forest plot of LE *versus* CC, outcome: ovulation rate per patient.

Despite including numerous studies with different populations and methods, moderate heterogeneity was seen among the trials (*I*^2^=57%), indicating some variability, though the overall effect was consistent across most studies. The forest plot shown in Figure 3 demonstrates that most individual trials favored LE, with most confidence intervals to the right of the no-effect line. Notably, large, high-weight studies such as Legro *et al*. (2014) and Ilangovan (2024) support the superiority of LE, adding strength to the results. Only a few studies, like Bansal *et al*. (2021) and Roy *et al*. (2012), slightly favored CC, but without statistical significance.

**Figure 4:**
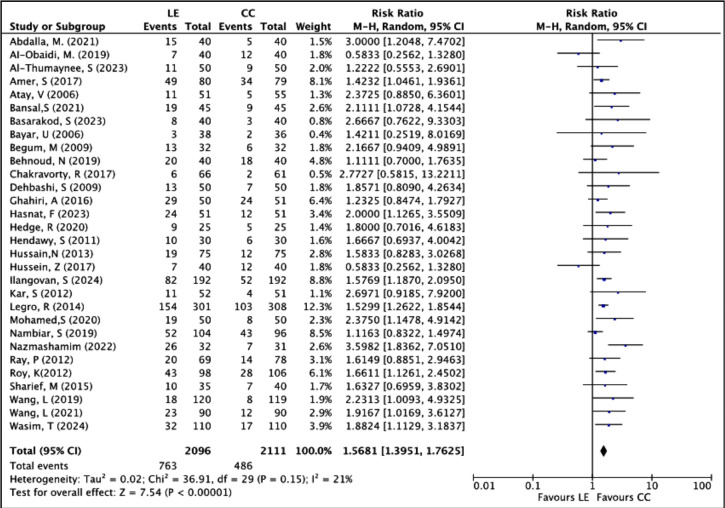
Forest plot of LE versus CC, outcome: pregnancy rate per patient.

Pregnancy rates per patient randomized were assessed in 30 RCTs, involving a total of 4207 women (LE group: 2096; CC group: 2111). There were 763 pregnancy events reported in the LE group, compared to 486 in the CC group. The pooled risk ratio (RR) indicated that patients treated with LE had a 57% higher chance of achieving pregnancy compared to those treated with CC (RR: 1.5681; 95% CI: 1.3951 to 1.7625; *p*<0.00001). The difference was statistically significant, and heterogeneity across studies was low (*I*^2^=21%), suggesting consistent results with minimal variation. The Chi^2^=36.91; df=29; *p*=0.15 also supports the absence of substantial heterogeneity. The forest plot shown in Figure 4 reveals that almost all individual studies favor LE, with most risk ratios positioned to the right of the line of no effect, and many CIs not crossing 1. Larger studies like Legro *et al*. (2014) and Ilangovan (2024), which carry significant weight, also support the superiority of LE.

**Figure 5:**
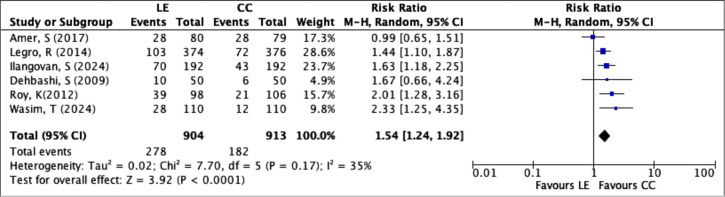
Forest plot of LE *versus* CC, outcome: live birth rate per patient.

Live birth rate was reported in 6 RCTs, involving a total of 1,817 women (LE group: 904; CC group: 913). A total of 460 live births occurred, with 278 in the LE group and 182 in the CC group. The pooled analysis showed a statistically significant difference in live birth rates between the LE and CC groups, with LE associated with a 54% increased chance of live birth compared to CC (RR=1.54; 95% CI: 1.24 to 1.92; *p*<0.0001), as shown in Figure 5. Heterogeneity among the studies was low (*I*^2^=35%, Chi^2^=7.70, df=5, *p*=0.17), indicating acceptable consistency across trials.

**Figure 6:**
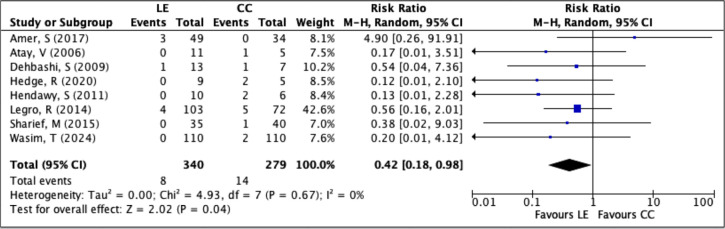
Forest plot of LE *versus* CC, outcome: multiple pregnancy rate per patient.

The multiple pregnancy rate was reported in 8 RCTs involving a total of 619 women (LE group: 340; CC group: 279). There were 22 multiple pregnancy events overall, with 8 in the LE group and 14 in the CC group. Meta-analysis showed that the risk of multiple pregnancy was significantly lower in women treated with LE compared to those receiving CC (RR=0.4229; 95% CI: 0.1834-0.9754; *p*=0.04). Heterogeneity among studies was negligible (*I*^2^=0%), indicating consistency across the trials (Figure 6). These findings suggest that LE is linked to a lower risk of multiple pregnancy than clomiphene citrate in infertile women, likely due to its more selective ovarian stimulation profile.

**Figure 7:**
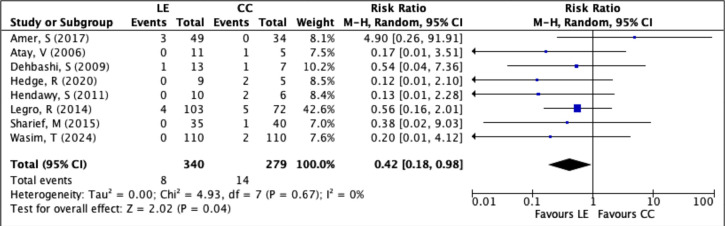
Forest plot of LE *versus* CC, outcome: miscarriage rate per patient.

The miscarriage rate per patient was reported in 11 RCTs, involving a total of 1866 women (LE group: 906; CC group: 960). There were 138 miscarriage events in total (LE group: 60; CC group: 78). This meta-analysis found no statistically significant difference in miscarriage rates between the two treatment groups. The pooled risk ratio (RR) was 0.9651 (95% CI: 0.7298 to 1.2764; *p*=0.80), suggesting a similar risk of miscarriage in both groups. Heterogeneity among studies was minimal (*I*^2^=0%), indicating consistency across individual trials. The forest plot shows that most included studies had wide confidence intervals crossing the line of no effect, further highlighting the lack of a significant difference between groups, as seen in Figure 7. Notably, the largest and most heavily weighted studies (e.g., Legro *et al*., 2014; Ilangovan, 2024) did not show a significant advantage for either drug. These results imply that the choice between LE and CC does not affect miscarriage risk and that both agents exhibit a similar safety profile in this regard.

## DISCUSSION

Most studies reported higher ovulation rates with LE compared to the CC group. In Amer *et al*. (2017), the ovulation rate was 83.8% for the LE group, *versus* 79.7% for CC. Similar results were found by Ilangovan (2024), with ovulation rates of 76.0% for LE and 55.2% for CC, and by Legro *et al*. (2014), who reported 88.5% with LE versus 76.6% with CC. Other studies, including Atay *et al*. (2006), Hasnat *et al*. (2023), Banerjee Ray *et al*. (2012), Ashfaq *et al*. (2018), and Nazmashamim *et al*. (2022), also supported this trend. Although Bayar *et al*. (2006) reported comparable or slightly higher ovulation rates with CC in isolated cycles, the overall evidence favors LE. The meta-analysis showed a relative risk (RR) of 1.18 (95% CI: 1.11-1.25) for this outcome, with moderate heterogeneity (I^2^=57%), confirming a statistically significant difference in favor of LE.

LE also showed significantly higher pregnancy rates. According to Amer et al. (2017), 61.2% of patients in the LE group achieved a clinical pregnancy, compared to 43.0% in the CC group. Ilangovan (2024) reported rates of 42.7% and 27.1%, respectively, while Roy et al. (2012) observed 43.9% for LE and 26.4% for CC. Even in smaller trials, such as Begum et al. (2009), the difference remained statistically significant (40.6% with LE versus 18.8% with CC). Although Behnoud et al. (2019) and Ghahiri et al. (2016) did not find significant differences between groups, both showed a tendency favoring letrozole. The meta-analysis reported an RR of 1.57 (95% CI: 1.39-1.76) with low heterogeneity (I^2^=21%).

Regarding live births, LE also demonstrated favorable results in the studies that included this outcome. Amer *et al*. (2017) found that 48.8% of women in the LE group had a live birth, compared to 35.4% in the CC group. Ilangovan (2024) reported rates of 36.4% *versus* 22.4%, and Roy *et al*. (2012) noted 39.7% for LE and 19.8% for CC. Legro *et al*. (2014) confirmed the trend in an extensive multicenter study, with 27.5% of live births in the LE group and 19.1% in the CC group. The overall statistical analysis revealed an RR of 1.54 (95% CI: 1.24-1.91) with moderate heterogeneity (I^2^=35%), supporting the conclusion that letrozole has superior efficacy for this outcome.

Regarding miscarriage, most studies showed similar rates between the two medications. For example, Amer *et al*. (2017) reported miscarriage rates of 18.4% with LE and 17.6% with CC. Similar data were found in Dehbashi *et al*. (2009), with 23.0% in the LE group and 14.3% in the CC group, and in Roy *et al*. (2012), who recorded 4.1% and 7.4%, respectively. Likewise, Legro *et al*. (2014) observed miscarriage rates of 31.8% with LE and 29.1% with CC. The meta-analysis confirmed no significant differences, with an RR of 0.97 (95% CI: 0.73-1.28) and no heterogeneity (I^2^=0%).

The risk of multiple pregnancy was consistently lower among women treated with letrozole. In the study by Hegde & Maitra (2020), none of the patients in the LE group experienced a multiple pregnancy, whereas 40% of pregnancies in the CC group were twins. This finding was echoed by Dehbashi *et al*. (2009), Ilangovan (2024), Kar (2012), and Nambiar (2019), all of whom reported no or significantly fewer cases of multiple gestation in the LE groups. The meta-analysis showed a clear benefit for LE, with an RR of 0.42 (95% CI: 0.18-0.97), confirming a statistically significant reduction in the risk of multiple pregnancies-an important advantage for maternal-fetal safety.

This review includes a broad and updated pool of evidence, synthesizing data from 33 randomized trials across various clinical endpoints. It is one of the most comprehensive comparisons to date between letrozole and clomiphene citrate for ovulation induction in PCOS. Unlike earlier meta-analyses that included fewer studies and smaller samples, this analysis provides greater statistical power and more precise estimates. Additionally, the inclusion of trials reporting not only ovulation and pregnancy but also live birth and miscarriage allows for a more thorough evaluation of both effectiveness and safety. The consistency of the findings across these outcomes strengthens the overall conclusion.

The findings of this review align with those of most individual studies included in the analysis. For example, Amer *et al*. (2017), Roy *et al*. (2012), and Ilangovan (2024) all reported better outcomes with LE. While one study (Bayar *et al*., 2006) showed ovulation rates slightly favoring CC in certain cycles, this was an exception and did not reflect the overall trend. Unlike some older meta-analyses that failed to show significant differences, likely due to smaller population sizes or variations in outcome reporting, this review benefits from a larger and more diverse data set, leading to clearer and more dependable conclusions.

From a clinical perspective, letrozole seems to offer several benefits over clomiphene citrate. Its higher rates of ovulation, pregnancy, and live birth, combined with a significantly lower risk of multiple pregnancy, make it a strong choice for first-line treatment in women with PCOS. Multiple pregnancies carry increased risks for both mother and baby. Therefore, a medication that lowers this risk without reducing effectiveness is especially important in reproductive medicine. Based on the positive benefit-risk profile shown in this analysis, letrozole should be considered a primary option in ovulation induction protocols.

Despite the strong results, some gaps still exist. For example, although miscarriage rates were reported in several studies, only a few addressed congenital anomalies, and the number of cases was too small to draw reliable conclusions. Additionally, most of the included trials took place in South and East Asia, with limited data from regions like Europe and North America. Future research should include more diverse populations to enhance generalizability. More studies comparing different dosing protocols and examining long-term outcomes, especially regarding child health, would also help guide safer and more effective clinical practices.

## CONCLUSION

This systematic review and meta-analysis demonstrates that LE is more effective than CC in several key clinical outcomes related to the treatment of infertility in women with polycystic ovary syndrome (PCOS). LE was consistently associated with a higher likelihood of ovulation, clinical pregnancy, and live birth, as well as a lower risk of multiple pregnancy compared to CC. These findings suggest that LE not only improves reproductive outcomes but also offers a safer profile in terms of pregnancy-related risks.

The miscarriage rate did not significantly vary between the treatment groups, suggesting that LE does not increase reproductive risk in this context. Despite some methodological differences among the studies, such as variations in dosage, cycle number, and study location, the consistent results across the main outcomes enhance the reliability of the evidence.

Therefore, LE is recommended as a first-line agent for ovulation induction in women with PCOS, especially given its superior clinical effectiveness and lower risk of multiple gestation. However, future studies should include more diverse populations, standardize treatment protocols, and evaluate long-term safety outcomes, particularly related to fetal development, to better support the use of LE as a preferred treatment option.
